# UHPLC-MS/MS method for the simultaneous determination of nicotine and tobacco-specific nitrosamines NNN and NNK for use in preclinical studies

**DOI:** 10.1007/s00216-022-04319-6

**Published:** 2022-09-26

**Authors:** Thomas Meikopoulos, Olga Begou, Theodoros Panagoulis, Eleni Kontogiannidou, Dimitrios G. Fatouros, John H. Miller, Georgios Theodoridis, Helen Gika

**Affiliations:** 1 Laboratory of Analytical Chemistry, Department of Chemistry, Aristotle University of Thessaloniki, 54124 Thessaloniki, Greece; 2 BIOMIC_Auth, Center for Interdisciplinary Research and Innovation (CIRI-AUTH), Balkan Center, Buldings A&B, 10th km Thessaloniki-Thermi Rd, P.O. Box 8318, 57001 Thessaloniki, GR Greece; 3 Laboratory of Pharmaceutical Technology, School of Pharmacy, Aristotle University of Thessaloniki, 54124 Thessaloniki, Greece; 4 Center for Research and Technology, Altria Client Services LLC, 601 E. Jackson Street, Richmond, VA 23219 USA; 5 Laboratory of Forensic Medicine and Toxicology, Medical School, Aristotle University of Thessaloniki, 54124 Thessaloniki, Greece

**Keywords:** LC-MS, nicotine, nitrosamines, TSNA, preclinical studies, porcine buccal epithelium

## Abstract

A new method was developed and validated for the simultaneous determination of nicotine and tobacco-specific nitrosamines (TSNAs) 4-(methylnitrosamino)-1-(3-pyridyl)-1-butanone (NNK) and *N*-nitrosonornicotine (NNN) in two different tests matrices: porcine buccal epithelium tissue and phosphate buffered saline (PBS) extracts of smokeless tobacco products. The novelty of this work is in the development of a liquid chromatography tandem mass spectrometry method that can provide simultaneous quantification of trace levels of TSNAs and high concentrations of nicotine in biological media. Precision, accuracy, and stability were evaluated during method validation to ensure the method was fit for purpose. Several sample preparation and extraction methods were evaluated to minimize matrix effects and maximize analyte recoveries. The method was accurate in the range of 81.1% – 117%; repeatability was estimated in the range of 1.5% – 13.6% across multiple concentrations. The linear regression correlation coefficient (*R*^2^) was greater than 0.9959 for all analytes, and the limit of detection (LOD) was determined for nicotine, NNK, and NNN at 1 ng/mL 0.005 ng/mL, and 0.006 ng/ mL, respectively. Our method was found to be appropriate for the analysis of nicotine, NNN, and NNK in the porcine buccal epithelium and PBS extracts of smokeless tobacco products.

**Graphical abstract:**

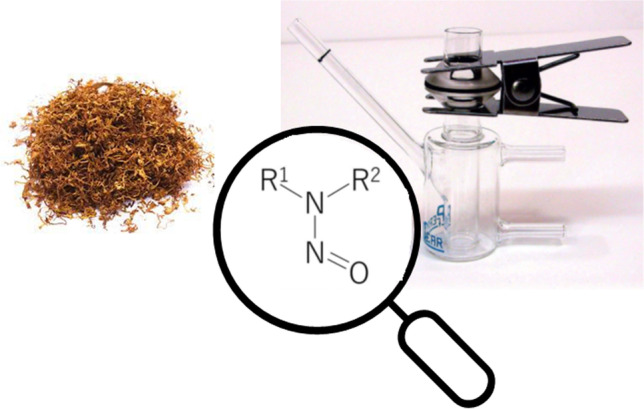

**Supplementary Information:**

The online version contains supplementary material available at 10.1007/s00216-022-04319-6.

## Introduction

**Fig. 1:**
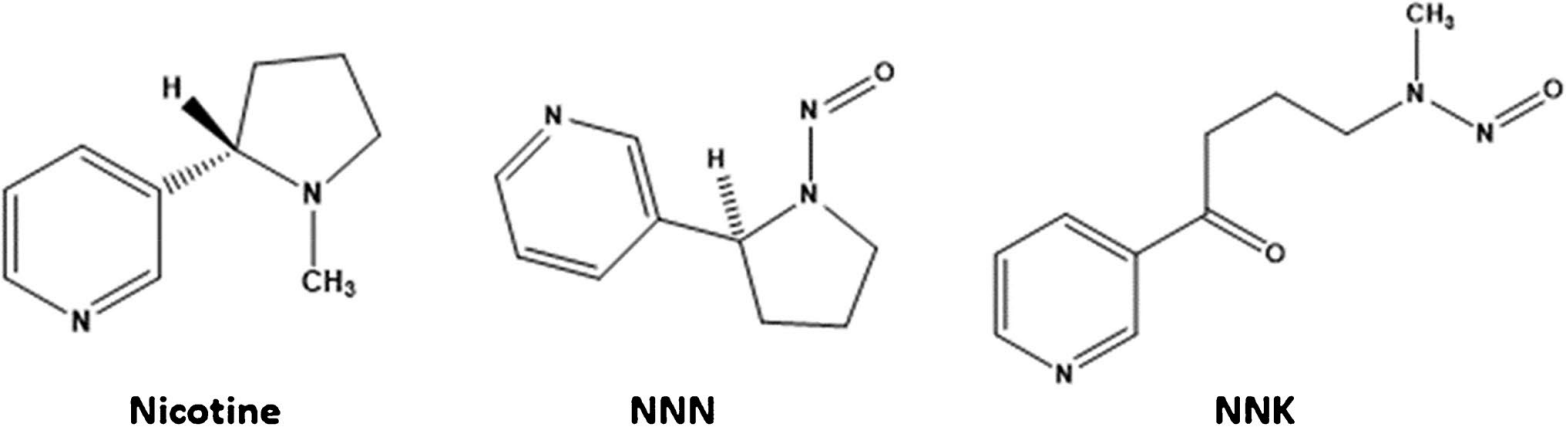
Molecular formula of nicotine, NNN and NNK

Nicotine and tobacco-specific nitrosamines (TSNAs) are compounds typically found in tobacco and tobacco smoke. Nicotine accounts for the 85-95% of the total alkaloid content in tobacco, whereas TSNA content is orders of magnitude lower in concentration. Some TSNAs have been identified as carcinogenic compounds [1, 2]. Among the TSNAs, 4-(methylnitrosamino)-1-(3-pyridyl)-1-butanone (NNK) and *N*-nitrosonornicotine (NNN) are of the highest concern in tobacco products. N-nitrosonornicotine (NNN) is produced during tobacco leaf ripening and processing. NNK is formed after tobacco harvest during curing, fermentation, and storage, when the pyrrolidine ring of nicotine opens converting nicotine into NNK [3, 4, 5]. The structures of 4-(methylnitrosamino)-1-(3-pyridyl)-1-butanone (NNK) and N-Nitrosonornicotine (NNN) are depicted in Fig. 1. The levels of NNK and NNN are largely dependent on the tobacco types used, agricultural practices, weather conditions, curing methods and manufacturing processes [5, 6], with additional amounts formed during smoking.

When tobacco is smoked, the TSNA compounds are absorbed, mainly though inhalation, into the bloodstream [7, 8]. In contrast, smokeless tobacco (ST) products, are consumed orally without combustion [9, 10], by chewing or by placing the product between the cheek or lip and gums [9]. Therefore, the TSNAs from ST products are primarily absorbed through the buccal mucosa. There are several methods reported in literature [11, 12, 13] to quantify TSNAs.

In order to characterize the uptake of tobacco constituents, via *in vitro* studies a robust analytical method is needed. Ideally such a method should measure multiple tobacco constituents in a single assay. Chromatographic techniques (LC, GC) combined with mass spectrometry (MS) [14] represent the method of choice for the quantification of nicotine or NNN and NNK in different matrices, such as urine [15, 16, 17], serum [18] , plasma [19, 20], tobacco [21, 22], smoke [23, 24, 25], e-Liquids [11, 12, 26] and indoor air [13]. However there appears to be only one report of a method that simultaneously quantifies nicotine, NNN, and NNK [22] by LC-MS/MS. This method was applied in tobacco leaves to determine high concentrations of the compounds, in contrast to the concentration ranges typically present in biological samples or preclinical *in vitro* studies.

Quantifying trace levels of TSNAs and nicotine in a single analysis is technically challenging because of the sizeable differences in their concentration ranges. Nicotine is the major alkaloid measured at concentrations ranging in the mg/g scale, while TSNAs are typically estimated in the ng/g scale. The scope of the present work was to develop a fast and efficient ultra-high pressure liquid chromatography tandem mass spectrometry (UHPLC-MS/MS) method capable of simultaneous quantitation of TSNAs and nicotine in a single run that could be potentially used in the evaluation of *in vivo* or *ex vivo* tissue studies. To the best or our knowledge such a method has not been reported so far in the literature. Our method exploits high sensitivity detection of TSNAs, while MS detuning of nicotine detection enables concurrent quantitative analysis in less than seven minutes.

## Experimental

### Materials and reagents

Methanol LC-MS grade was purchased from Fisher Scientific International, Inc. (Hampton, NH, USA). Ultra-pure water was collected by Milli-Q purification system (18.2 MΩ cm^-1^) (Millipore, Molsheim, France). Formic acid, 98-100% and ammonium formate ≥99% were obtained from Riedel-de Haën® (Sigma-Aldrich, Steinheim, Germany) and Fluka (Sigma-Aldrich, Steinheim, Germany) respectively. Ethyl acetate (EA) and methyl tert-butyl ether (MTBE) were purchased from the company Chem Lab (Zedelgem, Belgium), while potassium carbonate (K_2_CO_3_) from AppliChem PANREAC-ITW (Barcelona, Spain). NNK ≥99.6%, (1000 ppm), NNK-D4≥98%, (100 ppm), NNN ≥98%, (1000 ppm) and NNK-D4 ≥98%, (100 ppm) all pre-dissolved in acetonitrile (ACN) were obtained from SPEX organics (Metuchen, NJ, USA). Nicotine ≥99% and Nicotine-D4≥99% were supplied by Sigma-Aldrich, Steinheim, Germany. Fifteen milliliter centrifuge tubes with screw caps were obtained from BluCappTM, Nordhausen, Germany. Bond Elut Focus cartridges were purchased from Agilent, Santa Clara, United States. CORTECS C18 (2.1mm x 150mm, 3.5 μm), HSS C18 SΒ (2.1 mm x 100 mm, 1.8 μm), and BEH C18 (1.7 mm x 100mm, 2.1 μm) columns were obtained from Waters Corporation (Milford, MA, USA) Phosphate buffered saline (PBS) was freshly prepared every day (pH=7.4) NaCl, KCl, KH_2_PO4 and Na_2_HPO4 were purchased from Merck (Darmstadt, Germany). Porcine mucosal specimens, from a local slaughterhouse in Thessaloniki, were obtained from tissue removed from the inner cheek (buccal area) of freshly slaughtered pigs.

### Calibration standards and samples for quality control

Nicotine stock solution (1000 μg/mL) and stock internal standard solution (nicotine-D4) were prepared in methanol whereas NNN and NNK stock solutions (1000 μg/mL) and internal standards stock solutions (NNN-D4 and NNK-D4) were prepared in ACN. From the stock solutions, working standard solutions were prepared by dilution with a mixture of water and methanol 1:1 (v/v).

For the fortification of samples, a standard mixture of the studied compounds was prepared in water and methanol 1:1 (v/v) at 0.0362 μg/mL for NNN and NNK, and at 181.4 μg/mL for nicotine.

Calibration standards were prepared for two matrices tested, namely, PBS solution and tissue (porcine buccal) sample extracts (See details in study design section 2.4). More specifically, blank PBS solution was fortified at 0.785-, 1.56-, 3.13-, 6.25-, 12.5- μg/mL for nicotine, and at 0.156-, 0.312-, 0.625-, 1.25-, 2.5-, 5-, 10-ng/mL for ΝΝΚ and ΝΝΝ. Blank tissue extracts were fortified at 6.25-, 12.5-, 25-, and 50- μg/mL for nicotine; at 0.625-, 1.25-, 2.5-, 5-, 10-ng/mL for NNN; and at 0.04-, 0.08-, 0.15-, 0.31-, 0.625-, and 1.25- ng/mL for NNK.

Quality control (QC) samples were prepared at three concentration levels: low (LQC), medium (MQC), and high (HQC) by spiking standards respectively in PBS solution and in tissue extracts as a surrogate matrix for our studies. These QC samples were used to assess the precision and accuracy of the method. In more detail, LQC for PBS solution was prepared at 0.781 μg/mL for nicotine, 0.156 ng/mL for NNN and NNK; LQC for tissue extracts were prepared at 6.25 μg/mL for nicotine, 0.625 ng/mL for NNN, and 0.04 ng/mL for NNK. MQC for PBS solution was prepared at 3.125 μg/mL for nicotine, and at 0.625 ng/mL for NNN and NNK; MQC tissue extracts were prepared at 25 μg/mL, 2.5 ng/mL, and at 0.313 ng/mL for nicotine, NNN, and NNK, respectively. Finally, HQC for PBS solution was prepared at 12.5 μg/mL for nicotine, and at 2.5 ng/mL for NNN and NNK; and for tissue extracts HQC were prepared at 50 μg/mL, 10 ng/mL, and 1.25 ng/mL for nicotine, NNN, and NNK, respectively.

### Instrumentation and analytical conditions

The chromatographic separation was carried out on an ACQUITY UPLC H-Class System – (Waters Corporation, Milford, MA) on an Acquity BEH C18 column (150 × 2.1 mm i.d., 1.7 μm; Waters Corporation) protected by an Acquity BEH C18 VanGuard pre-column (5 mm × 2.1 mm i.d., 1.7 μm; Waters). The mobile phase consisted of A: 10 mM ammonium formate in water, pH=5.5 adjusted by 5M formic acid, and B: 10 mM ammonium formate in methanol, pH=5.5 adjusted by 5M formic acid. The applied gradient elution was as follows: 0-0.5 min: 1% B; 0.5-0.8 min: increase to 20% B; 0.8-1.0 min: increase to 55% solvent B; 1.0-3.0 min: increase to 100% solvent B; and finally at 3.01 min returned to the initial conditions and column was equilibrated for 4 min. The mobile flow rate was set at 0.45 mL/min and the oven temperature at 55^o^C. Injection volume was set at 5 μL.

MS/MS detection of analytes and internal standards (IS’s) was performed by a Xevo TQD system (Waters, UK) with electrospray ionization operating in positive mode. MRM mode was employed for the detection and quantification of nicotine, NNN, and NNK with the respective isotope labeled IS, nicotine-D4, NNN-D4, and NNK-D4 by observing the transition of the m/z of the precursor ion to quantifier product ion. For more accurate identification, qualifier product ions were also collected for the analytes. Optimization for cone voltage and collision energy were held for each analyte separately by direct infusion. Capillary voltage was set at +0.5 kV, source temperature at 500°C, while dessolvation gas flow was set at 1000 L/h and cone gas flow at 0 L/h. All parameters for the detection of the analytes are summarized in Table 1. Data acquisition and analysis were performed by MassLynx® (v4.1) software, while quantitation was accomplished by TargetLynx application.

**Table 1:** MS detection parameters for all analytes and IS

Analyte	Cone Voltage (V)	Collision Energy (V)	MRM transition
Nicotine	20	15	163.2→132.2*
		25	163.2→117.1
Nicotine-D4	30	15	167.2→136.1*
		25	167.2→121.1
NNN	25	10	178.2→148.3*
		28	178.2→119.2
NNN-D4	22	10	182.2→152.2*
		20	182.2→124.2
NNK	25	12	208.2→122.1*
		20	208.2→106.1
NNK-D4	22	12	212.3→126.2*
		20	212.3→110.1

*: quantifier ion

## Study design

### Tobacco extract preparation

An unflavored moist smokeless tobacco (MST), a CORESTA reference product (CORESTA CRP 2.1), was used for the method development and validation for nicotine and TSNA prior to *ex vivo* uptake studies using porcine buccal mucosa tissues.

One gram (± 0.0001 g) of each moist smokeless tobacco product was weighed in a 50 mL centrifuge tube. Twenty mL of Phosphate Buffered Saline (PBS) was added, and the vials were mixed on an orbital shaker for 30 minutes. The samples were then centrifuged for 10 min at 2680 g to remove all particulate matter. Sample supernatants were removed. The supernatant solution would typically be used as the donor phase in our *ex-vivo* studies, however for the purposes of developing and validating the method for these analytes the PBS tobacco extracts were further extracted as described under the extraction optimization section to assess the analyte recovery.

### Tissue preparation and ex vivo uptake studies

*Ex vivo* permeability studies provide a preliminary assessment of the carrier, thereby offering insights into the pathways and possible mechanisms of drug transport across the tissue epithelium. The permeability of the solutes across the oral mucosa was evaluated by means of permeability chambers. Typical permeability chambers (vertical Franz cells) are presented in Supplementary Fig. SI1.

Fresh porcine mucosal specimens obtained from a local animal processing facility were used as previously described [27]. The tissue was immediately removed and transported to the laboratory in ice-cold buffer (PBS) and laboratory processing commenced within 45 minutes (min). Scalpel and scissors were used to surgically remove excess connective and adipose tissue from the specimens. The mucosa thickness was measured to range from 760 to 1297 μm.

For our *ex-vivo* studies, appropriate sections of mucosa were mounted on vertical Franz type diffusion cells with a diffusion area of 4.9 cm^2^ and an acceptor compartment of 15 mL of PBS pH 7.4. After an equilibration period of 10 min with PBS on both sides, the acceptor compartment was filled with 15 mL degassed PBS pH 7.4 and the donor compartment with 2 mL of the tobacco extract. Traditionally, aliquots would be removed from the donor and acceptor phases at incremental times to measure the levels of these analytes in the two phases. In addition, post exposure, the treated porcine buccal tissues could be stored for further analysis of nicotine and TSNA.

## Extraction optimization

### Part 1

Both liquid-liquid extraction (LLE) as well as solid phase extraction (SPE) techniques were investigated to determine the sample extraction conditions which provided the best recovery and the cleanest extracts. For this evaluation we used the CRP2.1 MST sample extracted with PBS, which would be applicable for both the donor and acceptor phases from the Franz cell. For LLE, both MTBE and EA were tested at ratio 1:1 v/v solvent to sample. Samples were first adjusted to an alkaline pH using 5μL of K_2_CO_3_ (12M) and then extracted in the organic solvent. Basic pH is the optimal pH to extract these analytes from an aqueous extract since the basic functional groups will be neutralized, making the compounds more hydrophobic. Samples were vortexed for 2 minutes and then centrifuged to separate the aqueous and organic layers. The organic layer was transferred to a clean tube and evaporated till dryness under a stream of nitrogen. The dry residue was reconstituted with 700 μL of mobile phase A and was transferred into an autosampler vial for LC-MS/MS analysis. For SPE, Bond Elut Focus cartridges showing general application were used. The SPE cartridges were conditioned with 1 mL of methanol and 1 mL of water, following manufacturer’s recommendations prior to loading 1 mL of sample and adding 2 mL of H_2_O to remove matrix related components. The compounds of interest were eluted with 1 mL of methanol + 0.1% formic acid. The eluant was then evaporated till dryness under a stream of nitrogen. The dry residue was reconstituted with 700 μL of mobile phase A and transferred into an autosampler vial for LC-MS/MS analysis.

### Part 2

**Fig. 2:**
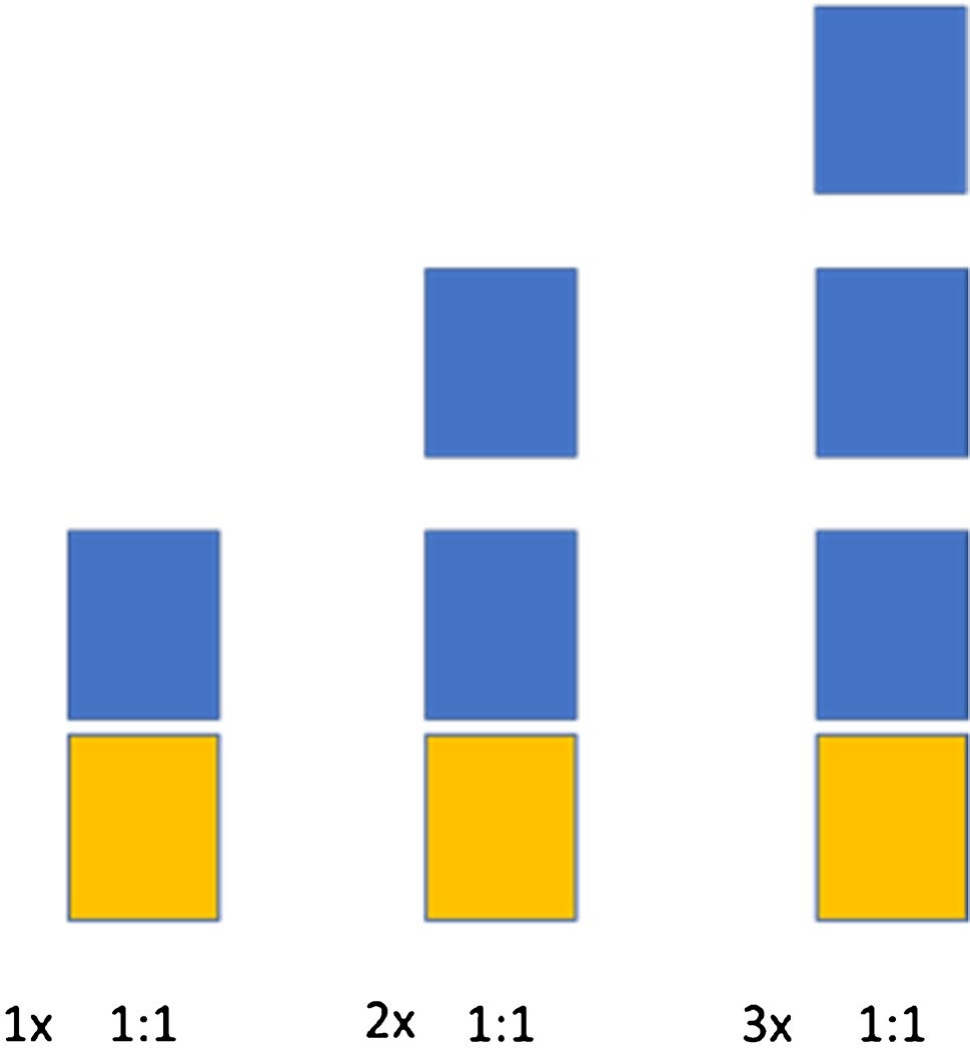
Schematic illustration of LLE extraction optimization protocol with ethyl acetate. Yellow blocks represent the part of the sample, while blue blocks represent solvent volume applied: one time (1 mL) (1x 1: 1), two sequential times (1mL+1mL) (2x 1:1) and three sequential times (1mL+1mL+1mL) (3x 1:1)

Following the optimization of the LLE solvent determined in section 2.5, additional optimization was conducted for the extraction of TSNAs, wherein each sample was extracted with an equal volume of solvent in one, two, or three steps, respectively (Fig. 2). More specifically, 975 μL of the MST extracted in PBS was transferred into a 15 mL centrifuge tube with screw cap. The pH of the solution was adjusted to 10 by the addition of 5 μL of potassium carbonate (12M) and then 20 μL of IS mixture 1 μg/mL. Sequential LLE were performed with 1 mL ethyl acetate each time. The mixture was vortex-mixed for 20 seconds and then centrifuged for 4 min at 3350 *g*. Eight hundred microliters of the organic supernatant of each step were combined in a clear Eppendorf tube and evaporated till dryness under a stream of nitrogen_._ The dry residue was reconstituted with 700 μL of mobile phase A and subsequently transferred into an autosampler vial for LC-MS/MS analysis.

## Tissue extraction optimization

The optimized extraction procedure for the tobacco extracts can also be applied to the tissue following this procedure. For tissue samples, a small patch of the tissue, equal to approximately 2 ±1 g, was cut and weighted in a vial, methanol was added in a 1:1 w/v ratio and the vial was placed in an ultrasonic bath for 1.5 hour at 25^°^C. The resulting extract was centrifuged at 6700 *g* for 10 min and filtered with 0.22 mm PTFE syringe filters. 200 μL of the clear supernatant were evaporated to dryness and the residue was reconstituted with 70 μL of methanol.

## Analytical method validation

The method was validated based on current regulatory guideline on bioanalytical method validation [28] including linearity, inter-day and intra-day precision, accuracy, sensitivity, selectivity and sample stability.

### Calibration

Calibration curves were prepared in different concentration ranges depending on the sample type as given in section 2.2. Each concentration level was analyzed four times and regression lines were established based on peak areas ratio of analytes (nicotine, NNN, and NNK) to that of their respective isotope labeled internal standards. LOD and limit of quantification (LOQ) were estimated experimentally, based on 3x signal to noise (S/N) and 10x S/N, respectively.

### Selectivity and carry over effect

Selectivity was established by analyzing blank tissue samples to detect any coeluting compounds.

Carryover from sample to sample was checked by analyzing blank methanolic samples after the HQC sample and the high concentration standard solution, at least twice for each batch of samples.

### Precision and accuracy

For the evaluation of intra- and inter-day precision and accuracy, QC samples containing nicotine, NNN, NNK at low, middle, and high concentration levels as described in section 2.2 in QC preparation, were analyzed in triplicate for each batch. Intra-day precision and accuracy were assessed with triplicate analysis performed in the same day; while for inter-day precision and accuracy, triplicate analysis was performed over a period of four consecutive days. The precision was expressed by % coefficient of variation (CV%) and accuracy as % experimental to nominal concentration.

### Extraction recovery

Extraction recovery of the analytes with the appropriate protocol as described above was assessed by analysis of blank samples spiked with the analytes before extraction at three concentration levels (LQC, MQC and HQC) and blank samples spiked with the analytes after extraction at the same concentration levels. Recovery was calculated by the percentage ratio of the mean peak area of samples fortified before extraction, to that of samples fortified after extraction.

### Stability

Stability was evaluated for samples kept in the autosampler for up to 72 hours(h), where temperature was set at 10°C. Stability was assessed by analyzing fresh and stored spiked quality control samples at low and high concentration levels and expressed as % change.

## Results and discussion

### Analytical method development and optimization

#### Instrumentation and analytical conditions

Three different analytical columns were evaluated to ensure optimum chromatographic separation of the three compounds (nicotine, NNN, and NNK), symmetrical peak shapes, and sufficient sensitivity. The three columns tested were a CORTECS C18 (2.1mm x 150mm, 3.5 μm) which is a solid-core particle that provides high resolution separation with lower back pressures, an HSS C18 SΒ (2.1 mm x 100 mm, 1.8 μm) which is a high strength silica column that can be used under high back pressure and a BEH C18 (2.1 mm x 100mm, 1.7 μm) column, which is a hybrid silica polymer based particle which typically provides high resolution separation with minimal peak tailing.

**Fig. 3:**
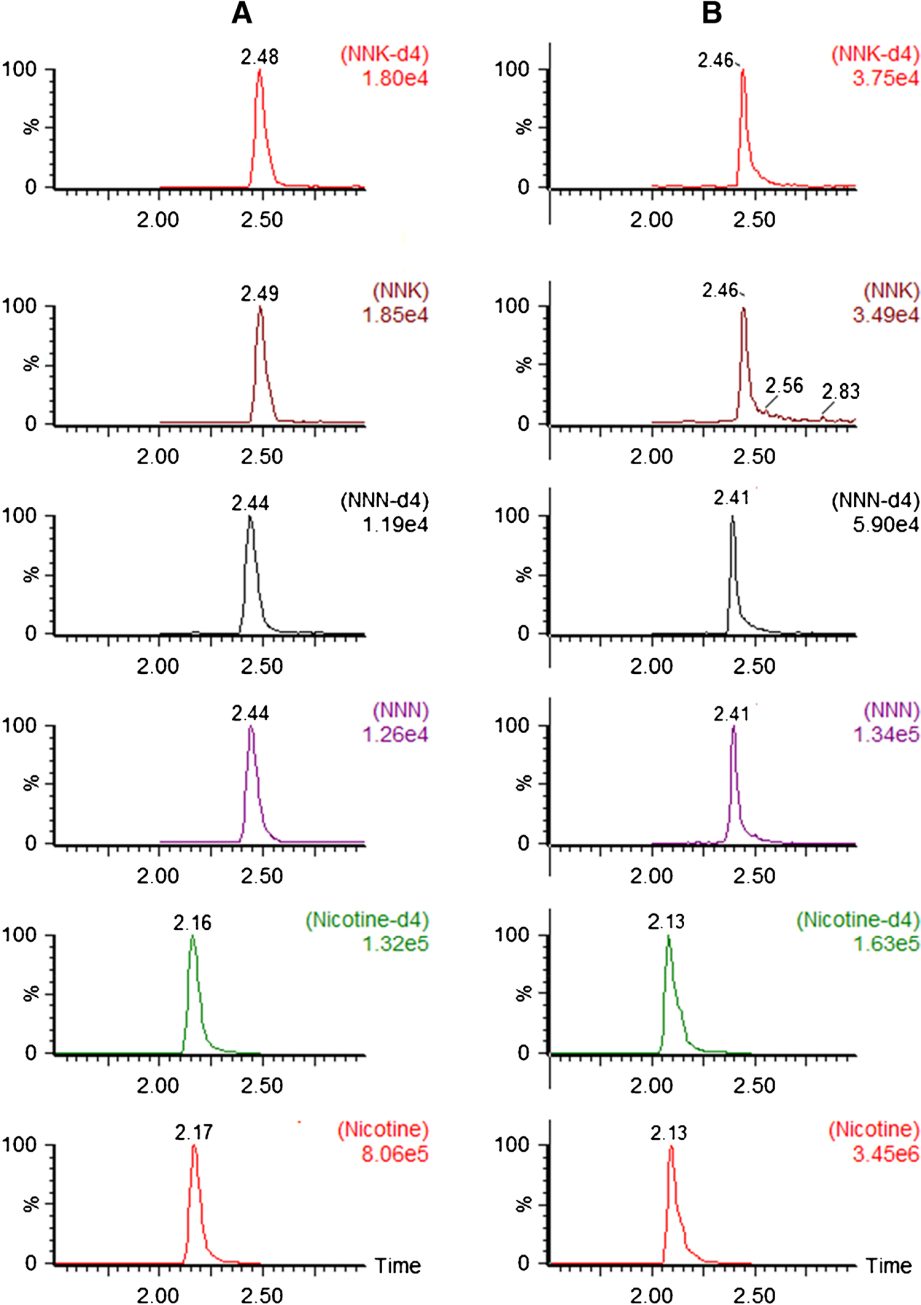
**A** LC-MS/MS chromatograms of nicotine, NNN, and NNK in spiked PBS sample at concentrations of 0.39 μg/mL for nicotine and 0.312 ng/mL for NNN and NNK; (**B**) real samples with 1.38 μg/mL nicotine, 0.54 ng/ml NNN and 0.28 ng/mL NNK

Various mobile phase compositions including mobile phase pH were evaluated to achieve optimum chromatographic performance. A water-methanol (H_2_O - MeOH) binary system acidified with 0.1% formic acid was first applied, whereas buffering with ammonium formate was also tested at various concentrations (5 mM, 10 mM, 15 mM). Injection volume, as well as column oven’s temperature were optimized in terms of peak shape and separation. The optimum chromatographic system was achieved with a BEH C18 column and a three-step gradient elution with increasing amounts of methanol compared water both buffered with 10 mM ammonium formate. Mobile phase’s pH value adjusted at 5.5 with the flow rate set at 0.45 mL/min provided the optimum peak shapes and peak separation of the three analytes in a 3-minute run. Figure 3 depicts representative chromatograms of all the analytes. Examples chromatograms for the other columns tested under similar conditions are provided in Fig. SI2.

For the mass spectrometry detection, cone voltage, collision energy, ionization polarity, precursor ion and product ion were optimized separately for each analyte in direct infusion mode. MS-MS product ions were selected based on the highest abundance. However, for nicotine, *m/z* 117.1 ion found to be more intense than *m/z* 132.2 as seen from the respective spectrum in Fig. SI3. To ensure that response of nicotine did not saturate the detector resulting in a non-linear response *m/z* 132.2 was selected as the quantification ion since it provided lower response than m/z 117.1 ion. Using an ion that has lower intensity for quantitation reduces method sensitivity for the specific analyte, avoiding saturation of the detector and the need for dilution and re-analyses of real samples.

The transitions that were selected for quantification were m/z 163.2 precursor ion to m/z 132.2 product ion for nicotine, m/z 167.2 to m/z 136.2 for nicotine-D_4_, m/z 178.2 to m/z 148.3 for NNN, m/z 182.2 to m/z 152.2 for NNN-D_4_, m/z 208.2 to m/z 122.1 for NNK and m/z 212.3 to m/z 126.2 for NNK-D_4_. The applied MS parameters are summarized in Table 1. Interestingly, it was observed that increased signal of the analytes was obtained with lower than ordinary capillary voltage values in combination to high source temperature, thus capillary voltage was set at 0.5 V and source temperature at 500°C.

#### Extraction optimization

**Fig. 4:**
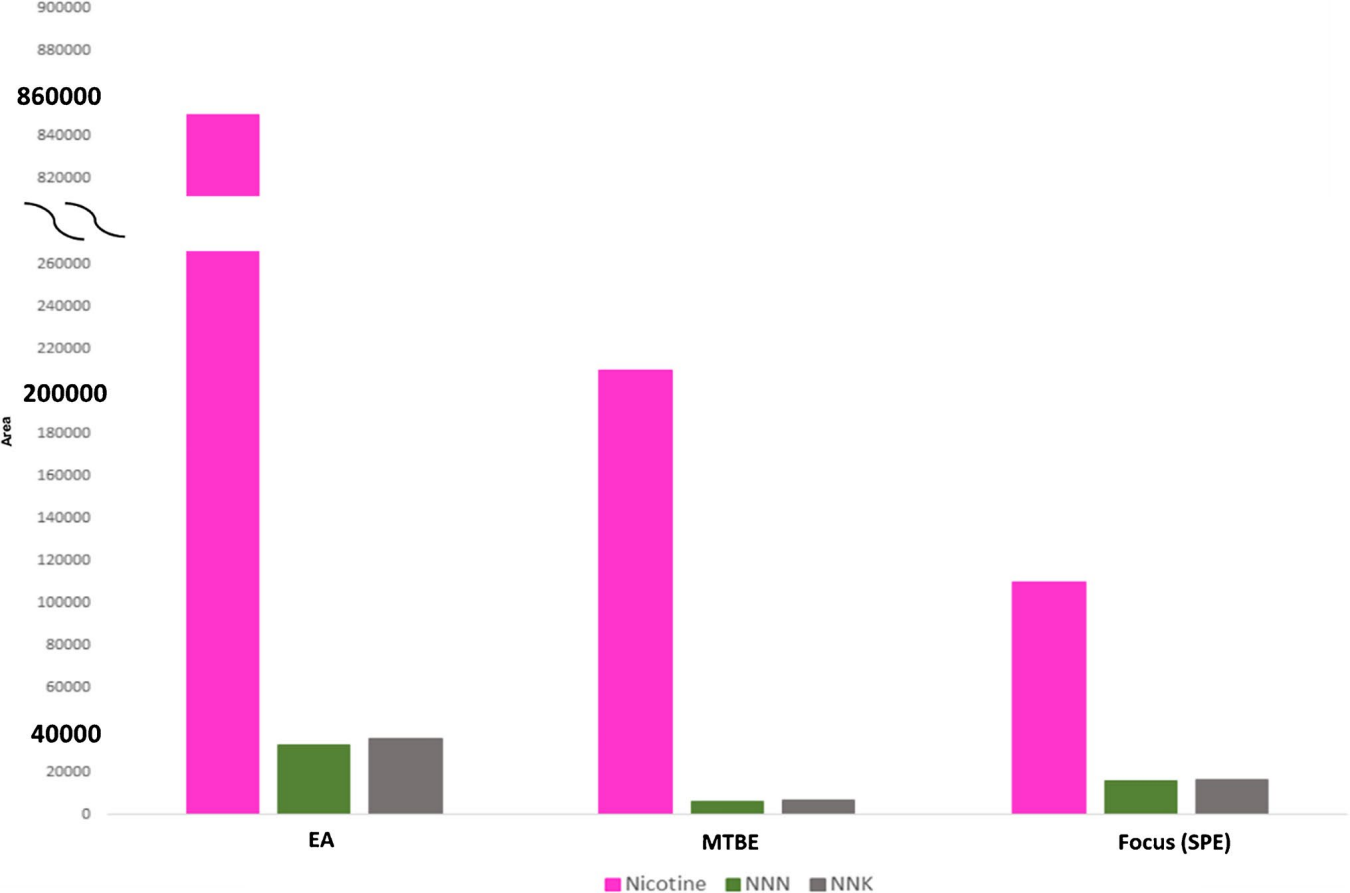
Bar plots showing peak area of the three analytes in different extraction tests: LLE with ethyl-acetate and methyl tert-butyl ether, solid phase extraction on Focus cartridges

Optimization of the extraction conditions using LLE and SPE was performed to maximize recovery of the analytes and to minimize any matrix related effects. Previous reported studies determining either nicotine or NNN and NNK perform SPE [12, 13, 18, 29]. Most of the cases have used C18 or Oasis HLB cartridges. Therefore, we attempted to use a general SPE protocol on a C18 material in accordance with published protocols. More specific, for applying the SPE protocol we used the Bond Elut Focus cartridges. As can be seen in Fig. 4, SPE showed similar results for all analytes with LLE protocol, when MTBE was used as extraction solvent. Thus, we determined that the SPE did not provide notable increase in extraction recoveries compared to MTBE LLE. When EA was used as LLE solvent, a remarkable increase in all responses was observed. In all cases, nicotine showed the highest performance, while NNN and NNK presented similar extraction behavior.

**Fig. 5:**
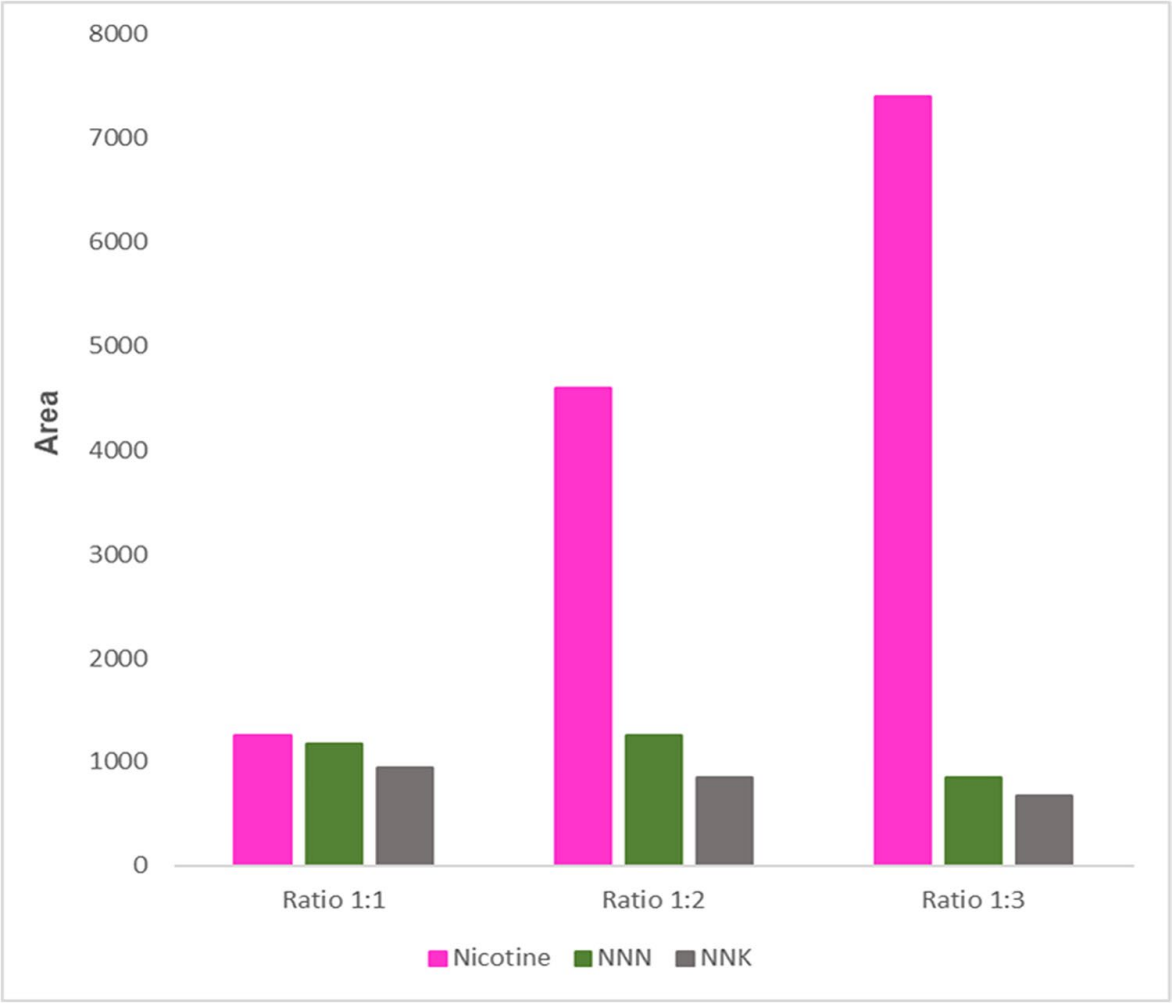
Bar plots showing analytes peak areas in different sample-to-solvent volume ratios (v/v) tested in liquid-liquid extraction of PBS with ethyl acetate

Therefore, LLE was chosen, as it allows for improved response and simpler and quicker treatment of the samples with. Alkaline pH obtained by the addition of saturated K_2_CO_3_ solution enhanced extraction. Extraction with EA showed higher response when compared to MTBE and was selected as the best solvent. As the nicotine peak was too intense, we performed a proper dilution for the next experiment. An evaluation of the sample-to-solvent ratio was conducted to optimize the recovery of the TSNAs. A sample-to-solvent ratio of 1:1 and 1:2 (v/v) provided the highest peak areas for NNN and NNK (Fig. 5). Although a solvent ratio of 1:3 (v/v) provided the best recovery of nicotine, the recoveries of TSNAs were lower in these samples. Since NNN and NNK are expected to be at trace levels in comparison to nicotine in the samples, and our method relied on labeled internal standards to track the recovery of the analytes, recovery and method sensitivity were heavily focused on these two analytes The final sample preparation was performed with two sequential LLE steps with 2 mL EA in total. Percentage recoveries of more than 71.6%, 82.1%, and 87.3% were calculated for nicotine, NNN, and NNK, respectively.

The method developed here is able to simultaneously quantify nicotine and TSNAs in low ng/mL levels in simulated preclinical sample matrices. To our knowledge, this is the first report on the simultaneous determination of nicotine, NNN, and NNK, suitable for the analysis of samples that contain trace levels of NNN and NNK as expected for *in vivo* studies. Previously reported LC-MS/MS methods that determined nicotine, NNN and NNK among other tobacco alkaloids and TSNAs [22] in tobacco leaf extracts exhibit application at ppm levels as the needs were for higher concentrations at these type of samples.

### Analytical method validation

#### Linearity and sensitivity

The method calibration exhibited linearity for PBS and tissue samples over different working ranges, which are presented in Table 2. Linearity was expressed by coefficient of determination (R^2^) of above 0.9957 for all analytes. The LOQ was found to be 3 ng/mL for nicotine, 0.02 ng/mL for NNN and 0.015 ng/mL for NNK. LOD was found to be 1 ng/mL for nicotine, 0.006 ng/mL for NNN and 0.005 ng/mL for NNK. The concentration range for nicotine was set to bracket the range of expected PBS samples from *invitro* studies.

**Table 2:** Linear range and R^2^ of the calibration curves, LOD-LOQ of the method

Analyte	Sample Type	Linear range	R^2^	LOD (ng/mL)	LOQ (ng/mL)
Nicotine	PBS	0.785 -12.5 μg/mL	0.9959	1	3
	Tissue	6.25 - 50 μg/mL	0.9986		
ΝΝΝ	PBS	0.156 – 2.5 ng/mL	0.9995	0.006	0.02
	Tissue	0.625 - 10 ng/mL	0.9957		
NNK	PBS	0.156 – 2.5 ng/mL	0.9974	0.005	0.015
	Tissue	0.04 – 1.25 ng/mL	0.9997		

#### Selectivity and carry over effect

Analysis of the blank tissue sample confirmed the absence of interfering, coeluting endogenous components. Carryover was estimated to be less than one percent of LOQ in the blank methanolic samples when analyzed after a HQC sample or a high concentrated standard solution, as described under the calibration and quality control section.

#### Precision and accuracy

Evaluation of accuracy and precision provided satisfactory results and found to be within acceptable criteria as seen from the data presented in Table 3. Intra-day precision was found to range between 1.5% and 12.7%, while inter-day precision between 2.1% and 13.6% for all analytes. Accuracy was found to range between 81.1% and 117%.

**Table 3:** Precision and accuracy for analytes

Analyte		Nominal concentration*	Concentration found					
			Intraday study			Inter day study		
			Mean ± s	CV%	Accuracy (%)	Mean ± s	CV%	Accuracy (%)
PBS	Nicotine	0.785 μg/mL	0.643 ± 0.03	5.1	82.3	0.63 ± 0.03	4.3	81.7
		3.125 μg/mL	3.366 ± 0.1	3.1	107	3.445 ± 0.1	3.0	110
		12.5 μg/mL	12.401 ± 0.2	1.5	99.2	12.289 ± 0.3	2.1	98.3
	NNN	0.156 ng/mL	0.156 ± 0.08	5.1	99.0	0.147 ± 0.02	13.6	94.2
		0.625 ng/mL	0.731 ± 0.04	5.1	117	0.71 ± 0.06	7.9	114
		2.5 ng/mL	2.355 ± 0.1	3.9	94.2	2.404 ± 0.08	3.3	96.2
	NNK	0.156 ng/mL	0.128 ± 0.02	11.7	82.1	0.125 ± 0.1	8.8	81.1
		0.625 ng/mL	0.705 ± 0.02	2.6	113	0.682 ± 0.03	4.8	109
		2.5 ng/mL	2.364 ± 0.12	5.0	94.6	2.487 ± 0.12	4.9	99.5
Tissue	Nicotine	6.25 μg/mL	6.365 ± 0.17	2.6	102	6.264 ± 0.29	4.7	100
		25 μg/mL	22.99 ± 0.48	2.1	92.0	23.74 ± 1.65	7.0	95.0
		50 μg/mL	52.05 ± 1.86	3.6	104	51.94 ± 1.42	2.7	104
	NNN	0.625 ng/mL	0.712 ± 0.05	7.0	114	0.695 ± 0.03	4.5	111
		2.5 ng/mL	2.442 ± 0.06	2.4	97.7	2.446 ± 0.05	2.0	97.8
		10 ng/mL	10.15 ± 0.61	6,0	101	10.125 ± 0.38	3.8	101
	NNK	0.04 ng/mL	0.045 ± 0.02	4.4	113	0.042 ± 0.003	7.1	105
		0.313 ng/mL	0.283 ± 0.03	11.3	90.4	0.281 ± 0.028	10.0	89.8
		1.25 ng/mL	1.24 ± 0.09	7.3	99.2	1.247 ± 0.073	5.9	99.8

(*n*= 9, three replicates per day for three days)

#### Stability

Stability tests were performed in spiked PBS solutions after approximately 72 h in the autosampler (10°C). Two concentration levels (LQC, HQC) were analyzed at time point zero (freshly prepared) and after being left for three days the autosampler. The results showed satisfactory stability with a percent (%) change 4.1% (LQC) and 3.4% (HQC) for nicotine, 9% (LQC) and 9.1% (HQC) for NNN, and 3.4% (LQC) and 3.5% (HQC) for NNK.

### Analysis of preclinical samples

The mean concentration of nicotine in the initial tobacco extracts were 201 ±41.5 μg/mL, while the respective donor phase mean value was 190 ± 20.5 μg/mL after the completion of the *ex vivo* exposures. The nitrosamines, NNN and NNK, were detected at much lower concentrations in tobacco extract and in the donor phase with mean levels of 57 ± 4 ng/mL and 24 ± 4 ng/mL, respectively. Finally, in the porcine mucosal tissue samples (~ 50 samples), the mean concentrations recorded were 11.3 ± 1.1 μg/g for nicotine, 0.002 ± 0.0002 μg/g for NNN, and 0.21 ± 0.03 ng/g for NNK.

## Conclusion

The newly developed method facilitates the concurrent determination of nicotine and tobacco-specific nitrosamines NNN and NNK in porcine buccal tissue samples. The procedure proved to be a reliable and fast method (7 min run) for the quantification of the analytes and was designed to determine compounds with widely divergent concentrations and orders of magnitude, ranging from 0.04 ng/mL to 50 μg/mL. The method met the acceptable criteria for accuracy and precision for all three analytes and was determined fit for purpose for the evaluated matrices.

## Supplementary information

ESM 1 (DOCX 1072 kb)

## Data Availability

Data sharing is not applicable to this article
