# Supplementary material for: UHPLC-MS/MS method for the simultaneous determination of nicotine and tobacco-specific nitrosamines NNN and NNK for use in preclinical studies
Source: Anal Bioanal Chem. 2022 Sep 26;414(27):7865–75. doi: 10.1007/s00216-022-04319-6 (PMC9568479; doi:10.1007/s00216-022-04319-6)
Supplement: Supplementary file 1 — (DOCX 1072 kb) [file 216_2022_4319_MOESM1_ESM.docx]

**UHPLC-MS/MS Method for the Simultaneous Determination of Nicotine and Tobacco-Specific Nitrosamines** **NNN and NNK for use in Preclinical Studies.**

Thomas Meikopoulos^1,2^, Olga Begou^1,2^, Theodoros Panagoulis^1,2^, Eleni Kontogiannidou^3^, Dimitrios G. Fatouros^3^, John H. Miller^4^, Georgios Theodoridis^1,2^, Helen Gika^2,5^*

^1^ Laboratory of Analytical Chemistry, Department of Chemistry, Aristotle University of Thessaloniki 54124, Thessaloniki Greece

^2^ BIOMIC_Auth, Center for Interdisciplinary Research and Innovation (CIRI-AUTH), Balkan Center, Buldings A&B, Thessaloniki, 10th km Thessaloniki-Thermi Rd, P.O. Box 8318, GR 57001

^3^ Laboratory of Pharmaceutical Technology, School of Pharmacy, Aristotle University of Thessaloniki, 54124 Thessaloniki, Greece

^4^ Center for Research and Technology, Altria Client Services LLC, 601 E. Jackson Street, Richmond, VA 23219, USA.

^5^ Laboratory of Forensic Medicine and Toxicology, Medical School, Aristotle University of Thessaloniki 54124, Thessaloniki Greece

**Corresponding author:* gkikae@auth.gr

**Supplementary Information**

**Figures SI**

**Fig. SI1** Schematic representation of Franz cell set up

**Fig. SI2** LC-MS/MS chromatograms for nicotine, NNN, NNK on the tested columns. (A: belongs to HSSC18 SD, 2.1 mm X 100 mm, 1.8 µm column test and B: belongs to cortex C18, 2,1mm Χ 150mm, 3,5μm column test

**Fig.SI3** Product ions mass spectra for all analytes (A: nicotine; nicotine-D4, B: NNN; NNN-D4, C: NNK; NNK-D4) in the optimal MS parameters


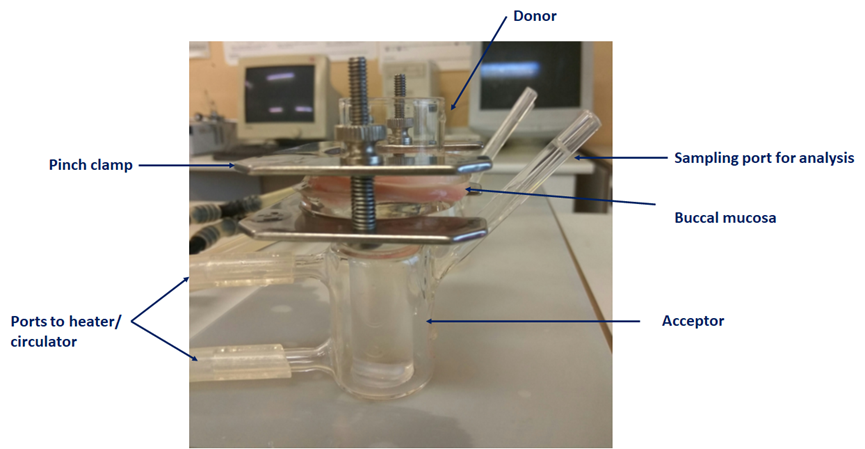


**Fig. SI1**


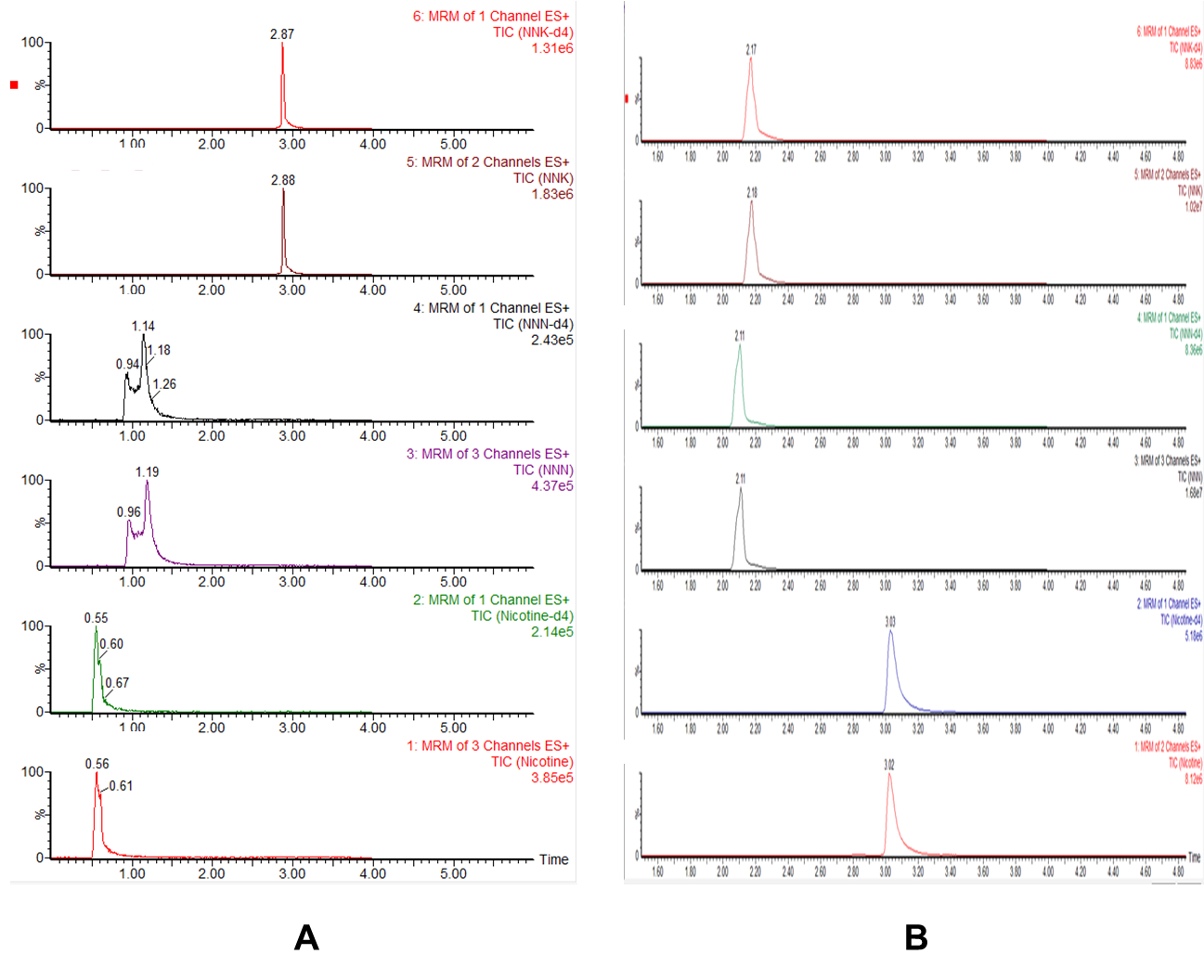


**Fig. SI2**


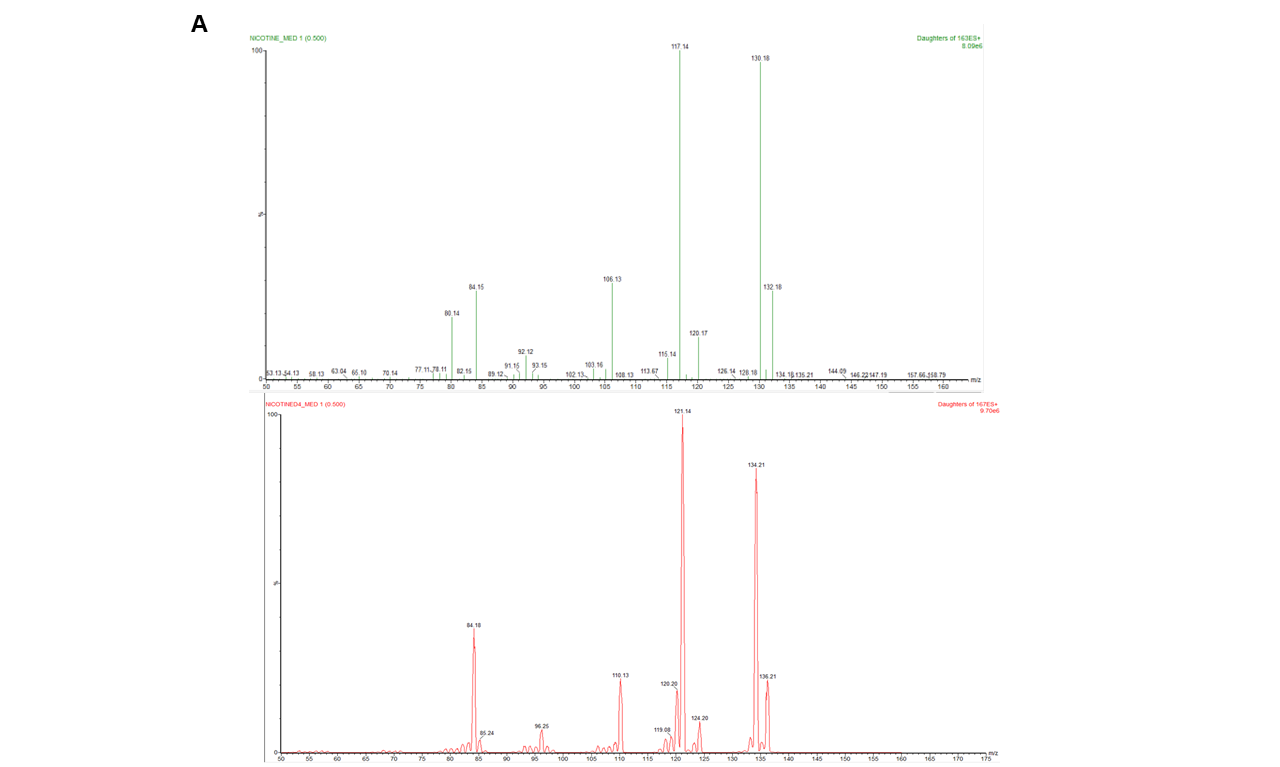

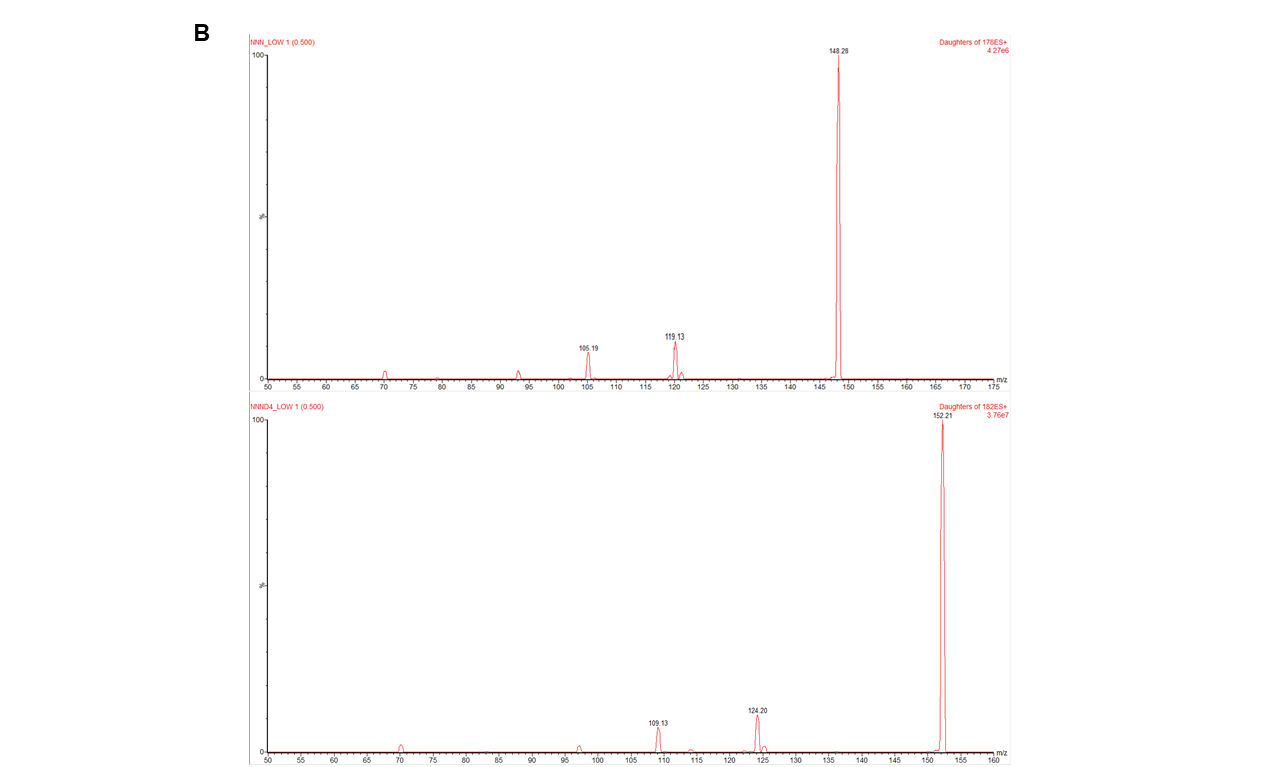


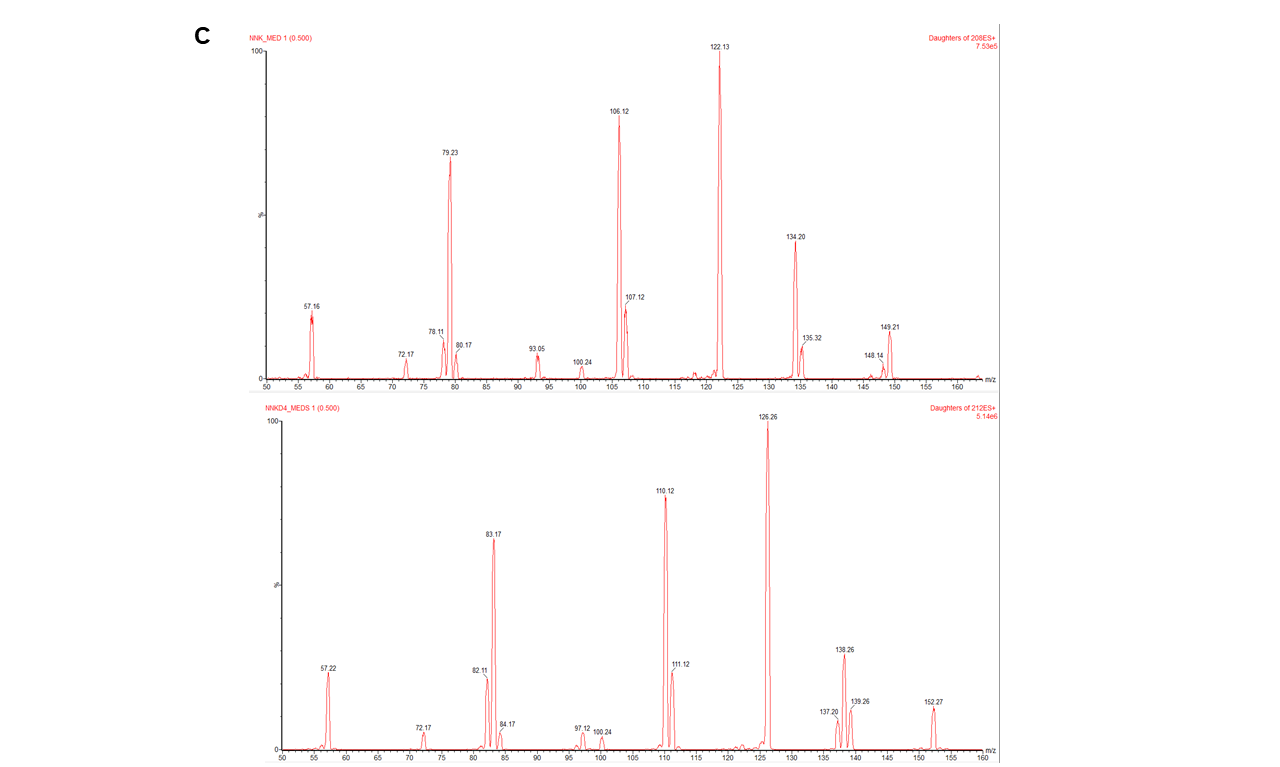


**Fig.SI3**
